# Systematic Identification of Caregivers of Patients Living With Dementia in the Electronic Health Record: Known Contacts and Natural Language Processing Cohort Study

**DOI:** 10.2196/63654

**Published:** 2025-05-05

**Authors:** Daniel Martin, Jason Lyons, J David Powers, Andrea E Daddato, Rebecca S Boxer, Elizabeth Bayliss, Jennifer Dickman Portz

**Affiliations:** 1 Institute for Health Research Kaiser Permanente Colorado Aurora, CO United States; 2 Department of Internal Medicine University of California Davis Sacramento, CO United States; 3 Department of Family Medicine University of Colorado School of Medicine Anschutz Medical Campus Aurora, CO United States; 4 Division of General Internal Medicine Department of Medicine University of Colorado School of Medicine Anschutz Medical Campus Aurora, CO United States

**Keywords:** caregivers, dementia, electronic health record, natural language processing, patient-centered care, patient portal

## Abstract

**Background:**

Systemically identifying caregivers in the electronic health record (EHR) is a critical step for delivering patient-centered care, enhancing care coordination, and advancing research and population health efforts in caregiving. Despite EHRs being effective in identifying patients through standardized data fields like demographics, laboratory results, medications, and diagnoses, identifying caregivers through the EHR is challenging in the absence of specific caregiver fields.

**Objective:**

Recognizing the complexity of identifying caregiving networks of people living with dementia, this study aims to systematically capture caregiver information by combining EHR structured fields, unstructured notes, and free text.

**Methods:**

Among a cohort of people living with dementia aged 60 years and older from Kaiser Permanente Colorado, caregiver names were identified by combining structured patient contact fields, that is, known contacts, with name-matching and natural language processing techniques of unstructured notes and patient portal messages containing caregiver terms.

**Results:**

Among the cohort of 789 people living with dementia, 95% (n=749) had at least 1 caregiver name listed in structured fields (mean 2.1 SD 1.1). Over 95% of the cohort had caregiver terms mentioned near a known contact name in unstructured encounter notes, with 35% having a full name match in unstructured patient portal messages. The natural language processing model identified 7556 “new” names in the unstructured EHR text containing caregiver terms among 99% of the cohort with high accuracy and reliability (*F*_1_-score=.85; precision=.89; recall=.82). Overall, 87% of the cohort had a new name identified ≥2 near a caregiver term in their notes and portal messages.

**Conclusions:**

Patterns in caregiver-related information were distributed across structured and unstructured EHR fields, emphasizing the importance of integrating both data sources for a comprehensive understanding of caregiving networks. A framework was developed to systematically identify potential caregivers across caregiving networks using structured and unstructured EHR data. This approach has the potential to improve health services for people living with dementia and their caregivers.

## Introduction

### Background

Around 27 million caregivers are assisting with self-care, household tasks, and health care management to high-need older adults [1], and nearly 15 million of these caregivers are assisting in the care of people living with dementia. Caregivers make up a diverse group of individuals that can include spouses, adults, children, family, and friends, who commonly provide personal care, medical-nursing tasks, run errands, maintain housekeeping, coordinate care across doctors or clinics, and offer financial assistance [2]. While many caregivers of people living with dementia find caregiving rewarding, caregiving is associated with negative health outcomes including increased chronic illness, depression, anxiety, stress, and mortality [3].

Systemically identifying caregivers in the electronic health record (EHR) is a critical step for delivering patient-centered care, enhancing care coordination, and advancing research and population health efforts in caregiving [4,5]. With improved caregiver identification, health care providers can involve them in decision-making processes, provide caregiving education, and offer resources and support to alleviate caregiver burden, ultimately improving communication and care coordination. This approach enhances the representation of caregivers who might not be recognized with conventional identification methods such as patient identification of caregivers or asking individuals if they consider themself to be a caregiver [6-8].

Despite EHRs being effective in identifying patients for health population management and health services research through standardized data fields like demographics, laboratory results, medications, and diagnoses, identifying caregivers through the EHR is challenging in the absence of specific caregiver fields. In response to the Caregiver Advise, Record, Enable Act, which requires hospitals to provide an opportunity for patients to self-identify a caregiver, efforts are in place across the United States to standardize caregiver EHR fields, in addition to “health proxy” and “emergency contact” elements [9]. It is important to note that individuals listed in these structured fields are not always caregivers and are therefore termed here “known contacts.” Despite improvements, standard caregiver fields are not widely available, and the collection of multiple caregivers is even more limited. In 2019, there was a 50% chance that hospital staff asked the patient to identify a family caregiver, and even if asked not all patients chose to identify a caregiver [10]. Although the implementation of the CARE Act requirements may support caregiver identification, the policy is limited to hospitalizations such that detailed documentation of the full caregiving network will not always be available in ambulatory care settings.

We previously developed a rule-based algorithm to identify people living with dementia and caregiver dyads linked by structured data, including insurance information and residence [11]. However, our previous model was limited to caregivers living in the same household, often spouses, who share health insurance. While caregiver identification is limited by formal documentation in structured caregiving fields, EHRs often have a wealth of unstructured caregiving data in clinical notes and patient portal messages. Natural language processing (NLP) applied to clinical documentation may allow for systemic identification of caregivers across the caregiving network. Leveraging unstructured EHR data to find documentation patterns referencing caregivers will allow us to further identify caregivers outside the home (eg, adult children and extended family members), nontraditional caregivers (eg, friends and neighbors), and those not listed in formal, structured fields. Merging structured and unstructured data has previously been used to improve clinical prediction models [12] in comparison to structured data-only models and unstructured data-only models for emergency care [13] and mortality [14].

### Objective

The primary objective of this research was to develop a framework for merging structured known contact fields with unstructured EHR text to systemically identify caregivers of people living with dementia who may benefit from caregiver and dyadic interventions.

## Methods

### Study Design, Setting, and Population

**Figure 1:**
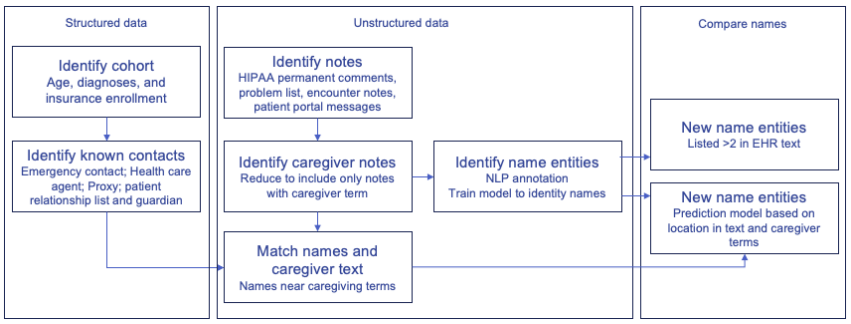
Overview of the data flow used for data extraction, NLP, and name-matching to identify care partners of people living with dementia. HER: electronic health record; HIPAA: Health Insurance Portability and Accountability Act; NLP: natural language processing.

We conducted a retrospective cohort study at Kaiser Permanente Colorado (KPCO), an integrated nonprofit health care delivery system, to systemically identify caregivers of people living with dementia by analyzing structured known contact fields and unstructured EHR text. We identified 789 people living with dementia with behavioral disturbances, as defined by Multimedia Appendix 1, aged 60 years and older, enrolled in KPCO from January 1, 2020, to November 02, 2022, with one or more in-person or telehealth visits after the initial *ICD* (*International Classification of Diseases*) code was added to the patient problem list. People with advanced dementia often require assistance with nearly all activities of daily living (eg, feeding, dressing, and toileting) [15] and exhibit complex behaviors such as agitation or resistance to care [16]. Therefore, as an approach to identify patients who were more likely to need a caregiver and have a caregiver documented in their medical records, we included behavioral disturbance codes. We planned to include patients aged 65 years and older aligned with Medicare enrollment; however, to maximize data availability to train the NLP model, we expanded age inclusion to 60 years. To identify the caregiving network, this research took place over three phases: (1) identification of known contacts from structured EHR data fields, (2) identification of unstructured notes and patient portal messaging that include caregiving text, and (3) identification of names of potential caregivers that are not listed as known contacts from caregiving text (Figure 1).

### Ethical Considerations

This research was reviewed and approved by the KPCO institutional review board (1956520). A waiver to obtain informed consent and a waiver to obtain Health Insurance Portability and Accountability Act (HIPAA) privacy rule authorization were approved. Identifiable data was only accessible by study team members trained in the ethical conduct of research using institutional review board–approved protocols including KPCO data security measures. No compensation was provided for this study.

### Data Sources and Variables

#### Overview

This exploratory work used 2 primary data sources including the KPCO and the Virtual Data Warehouse [17]. The Virtual Data Warehouse was used to establish the cohort of people living with dementia and the EHR was used for collecting structured patient contact data (ie, known contacts) and unstructured patient notes and patient portal messages.

#### Structured Data Extraction of Potential Caregivers—Known Contacts

The first phase of this research was to identify potential caregiver names in structured fields of the EHR. Structured fields in the EHR can contain names and contact information for emergency contacts, health care agents (people designated to make health care decisions when a patient is incapacitated), and legal guardians for people living with dementia, who are likely to provide some type of support in some capacity. Again, as the listed individuals are not always caregivers, they are termed here as “known contacts.”

#### Unstructured Data Extraction Related to Caregiving

The study’s second phase was to identify unstructured text fields within the EHR, that may contain the names of known contacts and additional names of potential caregivers not explicitly listed in structured known contact fields. Unstructured fields included the following locations: HIPAA permanent comments, problem lists, encounter notes, appointment notes, hospital notes, and messages sent using the patient portal. Messages sent using the patient portal included both messages sent from the people living with dementia’s account and messages sent from a registered proxy portal account associated with the people living with dementia’s account. Of note, only 11% (n=88) of our cohort of people living with dementia had an associated proxy portal account. To aid in the identification of text related to caregiving and listed caregiver information, we used a list of caregiver key terms developed by Mahmoudi et al [18]. We modified the list to remove any terms of formal paid caregivers (eg, nurse, aide), included the terms “in law” and “carer,” and added common abbreviations such as “DIL” for daughter-in-law and “dtr” for daughter. However, the acronyms DIL, BIL, and SIL were ultimately removed from the caregiver term list due to conflicts with common medical procedures. A full list of caregiver terms is listed in Multimedia Appendix 2. The unstructured EHR text strings with caregiver terms were concatenated into a single string with a limit of 2^15^ characters. Of the 658,809 unstructured text strings found for the cohort, 38 were over this character limit and excluded. This unstructured EHR text from the notes and portal messages containing caregiver terms is referred to as “caregiver text.”

### Matching Known Contacts and Caregiver Text

We then programmatically cross-referenced the known contact names with caregiver text notes. This allowed us to determine which note listed a known contact next to a caregiver term. For example, we can see “John Smith” is listed as an emergency contact, and he is also listed as “patient’s son” in appointment notes, and he sent a portal message on behalf of the patient signed by “John.” We used PRX Perl (SAS Institute) regular expression features, a software program in SAS (version 9.04; SAS Institute, Inc) to review the 5 words before and after a caregiver term to match names of known contacts within proximity of the key term. Because names can be misspelled or abbreviated, we used an approximate matching algorithm to identify matching names. We deployed the SPEDIS (spelling distance) function in SAS which computes a generalized edit distance between 2 strings optimized for spelling differences between individual words. Larger SPEDIS scores indicate greater differences between the 2 words. We matched each word in a text string against the names of known contacts and kept the closest match within the threshold of a cutoff score found in testing to match simple spelling errors while minimizing false positives. Our cutoff accepted spelling differences of 1 letter replacement or addition, or 2 pairs of letters with their positions swapped. We flagged matching first names and matching first-last name pairs. To confirm this matching methodology and validate the data, 2 study team members (JDP and AED) looked at a random sample of 100 notes to manually identify caregivers. Half of the validation notes came from unstructured text where the matching algorithm identified the name of a known contact and half came from unstructured text where no known contact was identified by the algorithm. The 2 study team members looked for names (first name and last name) and caregiver terms. For accuracy and consistency, JDP and AED reviewed 25 notes together to discuss the review process. The final 75 were completed independently by AED. Issues with data, such as misspelled names, were discussed as a team. 100% of the text samples with known contacts identified by the algorithm (n=50) were verified to contain names of known contacts. Of the text sample without identified known contacts (n=50), 1 note (2%) was manually verified to contain the name of a known contact, and 2 notes (4%) contained references to possible caregiver names that were not matched to known contacts.

### NLP to Identify Names of Caregivers Not Listed as Known Contacts

To also find names of individuals who may be caregivers who are not listed as a known contact, our final phase of the study used NLP to identify caregiver names within the caregiving text. Using the same approach for manual review of name matching previously discussed, a team member (AED) first manually annotated a random sample of 100 full-text notes from multiple sources (permanent comments, portal messages, and problem list), where 50 notes included caregiver names and 50 did not. The annotations in this first pass included multiple entity categories for names, email addresses, and phone numbers occurring in the text. To distinguish caregiver names, phone numbers, and email addresses from other names, we labeled the following entity types: caregiver name, caregiver email address, caregiver phone number, other person name, other person email address, other person phone number, and provider name. After these annotations were made, we updated spaCy’s large English language web-based pipeline by adding the new entity types and updating this pipeline through spaCy’s training functions, using 100 training iterations. After manual inspection by JDP and a review of examples by the team from the entity predictions from the updated model on a separate sample of full-text notes, we decided the entity scheme was too complicated, especially for the small amount of data being used, and determined that the updated entity recognition model was not performing well.

To simplify the entity scheme, 4 study team members (AED, JDP, DM, and JL) annotated a random sample of 200 full-text palliative care notes for a single-person entity. We focused on palliative care notes based on frequencies when searching for caregiver terms and known contacts in text, and the clinical significance of the encounter where those terms and names are found. In this case, the annotation team identified any name of a person (caregiver, patient, and provider) in the text and tagged it as a person entity. Email addresses and phone numbers were not included in this round of annotation. Before annotation, the full team met to review the annotation process and requirements. To ensure we were identifying names that were unique, we annotated text that appeared to be first names, full names, and nicknames. After a week of annotation, issues with name entity annotation, such as medications that looked like person names, were discussed and addressed as a team. The team reviewed annotation questions or concerns weekly until the annotation was complete. The training was then conducted in spaCy using a blank language model, only using the tokenizer and a convolutional neural network with 4 layers using 4 words on either side of each word token. This model was then used to predict person entity tags in the full-text notes from the full dataset from all sources for further processing in SAS.

Language models and training were conducted in Python (version 3.11; Python Software Foundation). Text annotation was conducted in Prodigy (version 1.14.8; Explosion AI). NLP training was conducted and pretrained language models were acquired from spaCy (version 3.7.2) [19]. We discussed using advanced language models such as a transformer-based deep learning model that has been pretrained on large datasets. Transfer-based models are highly effective at capturing complex linguistic patterns, enabling them to achieve higher accuracy across a wide variety of NLP tasks compared to traditional models. However, it requires significant computational resources (eg, high-performance CPUs and GPUs) and larger datasets for fine-tuning. Additionally, while it is a powerful model, it can sometimes lack interpretability compared to spaCy, which is optimized for speed, efficiency, and easier deployment in production environments. The evaluation of the model’s performance included an *F*_1_-score, accuracy, sensitivity, and specificity. *F*_1_-score is a measure of an algorithm’s predictive power, combining precision, and recall. Accuracy gauges the correct classification of both positive and negative observations based on manual annotations of the notes.

Identified names were then programmatically cross-referenced with the first or full names of known contacts, the people living with dementia’s name, and all known providers from each person living with dementia’s health care encounters within the past 3 years to exclude matches to these names. The name text was also scrubbed of common English words and placed names that were sometimes erroneously included by the spaCy model along with person names. The remaining identified names were then flagged as “new” names in addition to known contacts.

The structured and unstructured data were merged into the final dataset at the caregiver level. For each caregiver identified, the final merged dataset included a caregiver ID, the name of the associated patient from the chart as receiving care and the patient’s study ID, the caregiver’s first name, the caregiver’s full name, a binary indication of “yes” or “no” as to whether their name was listed in each of the structured fields, a binary indication that the caregiver was flagged as a “new name,” the number of times their name was located in each type of caregiving text medical note, and caregiving terms associated with their name. It is important to note that we had little missing data; 95% of the cohort had at least 1 known contact and 99% had at least 1 string of caregiving text to analyze. We made the assumption that missing data indicated that the patient did not have a caregiver documented.

There were several challenges to merging the structured and unstructured data. First, while names in structured fields were in a specific format, names in the unstructured data could be nicknames, misspelled, or abbreviated. Therefore, we chose a fuzzy matching cutoff score to account for minimal errors and matched based on first only and full. Second, some patients had multiple caregivers listed in several locations, and we needed to identify and remove duplicates. The relationship type from structured data was standardized by drop-down options, but unstructured data were difficult to classify. An example is the term “daughter” in 1 clinical note may also refer to the same name identified with the term “mother” from a message. Since we were unable to decipher these classifications at this time, we broadly categorized them in a “child key term” category. Finally, recurrent documenting in EHR templates and repeated copying and pasting of clinic notes may overestimate the frequency of certain caregiver names.

To increase precision for identifying names of individuals who are likely to be caregivers we examined unique “new” names identified from the spaCy model that were listed two or more times in the EHR caregiving text. We also explored a prediction model using logistic regression to identify unique names with patterns in their occurrence in EHR caregiving text that are similar to the patterns observed among names of known contacts. Each known contact and newly identified name occurring at least once in EHR caregiving text was tabulated with the number of times that name occurred in each EHR text source and the number of times that name occurred next to caregiver terms grouped into categories of relationship types. Those variables were used in a logistic regression model to predict if a name was a known contact or new. After variable selection, binary variables rather than counts were used for the presence (yes or no) of a name in encounter notes, HIPAA permanent comments, problem lists, and portal messages, and the presence of a name next to a spousal key term, child key term, or any other caregiver term. The predicted probability of a case being a known contact was extracted from this model, and new names were identified that had a predicted probability above the 10th percentile of predicted probabilities for known contacts.

## Results

### Matching Known Contacts and Caregiver Text

#### Identification of Known Contacts

The 789 people living with dementia were on average 84 years of age, 62% female, primarily White (80%) and 11% were Hispanic. Among the cohort, 749 (95%) of them had at least 1 caregiver name listed as a known contact (Table 1). The 1667 known contacts listed in structured fields included: emergency contact (44%), health care agent (30%), legal guardian (3%), portal proxies (5%), and close relative or friend listed in the general patient contacts table (97%). There was a large degree of overlap between the names found in these sources.

The cohort had a median of 2 known contacts per person living with dementia (mean 2.1, SD 1.1). While not a standard field, we found that 53% of the cohort (n=418) had an individual listed in the “transportation” flowsheet. Known contacts listed in the general patient contacts table were most often a child, other relationship, or spouse, and 47% of known contacts shared the same last name with the patient (Table 2).

**Table 1:** Demographics of patients living with dementia.

					People living with dementia cohort (N=789)			People living with dementia with contacts or relations recorded (N=749)
**Demographics**								
	Age (years), mean (SD)			84.3 (8.1)			84.3 (8.1)	
	Sex (female), n (%)			492 (62.4)			474 (63.3)	
	**Race, n (%)**							
		American Indian, Alaskan Native, Pacific Islander, or Native Hawaiian	12 (1.6)			11 (1.5)		
		Asian	16 (2)			14 (1.9)		
		Black or African American	29 (3.7)			28 (3.7)		
		Hispanic	88 (11.2)			81 (10.8)		
		White	627 (79.5)			601 (80.2)		
		Other^a^	60 (7.6)			53 (7.1)		
		Unknown or not reported	45 (5.7)			42 (5.6)		
**Contacts or relationships in EHR^b^**								
		Contacts or relationships, mean (SD)	—^c^			2.2 (1.1)		
		Has any contact or relationship, n (%)	—			749 (100)		
		Has relationship recorded, n (%)	—			749 (100)		
		Has emergency contact, n (%)	—			737 (98.4)		
		Has guardian, n (%)	—			56 (7.5)		
		Has health care agent, n (%)	—			297 (39.6)		
		Has portal proxy, n (%)	—			78 (10.4)		

^a^Mixed race, Middle Eastern.

^b^EHR: electronic health record.

^c^Not applicable.

**Table 2:** Sources and relationship type for known contacts (N=1667).

Known contacts from structured fields		Values
**Contact type, n (%)**		
	General patient contacts	1610 (96.6)
	Emergency contacts	737 (44.2)
	Legal guardians	56 (3.4)
	Health care agents	502 (30.1)
	Portal proxy	89 (5.3)
**Relationship type**		
	Child or parent	903 (54.2)
	Spouse	260 (15.6)
	Other	285 (17.1)
	Unknown	219 (13.1)
	Same last name as patient	785 (47.1)

#### EHR Caregiving Text

Almost all people living with dementia had a caregiver term listed in the unstructured EHR text. Among the encounter notes text (N=662,536), caregiver terms were found in 26% of cases (n=172,259). The success rate for identifying caregiver terms was 34% in the patient portal message text (N=34,504) and 14% in the problem list text (N=24,948). While permanent comments provided the lowest volume of text (N=781) a caregiver term was listed in 99% of the text. Child-related caregiving terms were the most frequently identified caregiver terms in the permanent comments (52%), encounter notes (9%), and the problem list (5%), but parent-related caregiving terms (22%) were the most common in the patient portal messages. Other family (13%) and spouse-related (27%) caregiving terms were also commonly found in permanent comments.

#### Location of Known Contacts in EHR Caregiver Text

Among the 749 who had at least 1 known contact, when we matched caregiver term text with known contacts (first name and full name; N=1667), 94% of the cohort had at least 1 mention of a known contact’s first name (n=1567), 88% of a full name (n=1467) near a caregiver term in encounter notes, and 35% had a full name match in patient portal messages. Among known contacts, 74% had their full name mentioned at least once in an encounter note, 55% were listed alongside a child keyword, and 19% were listed in a patient portal message. A median of 2 (IQR 1-3.5) known contacts per person living with dementia had their full name found near a caregiver term in at least 1 encounter note and a median of 1 known contact per person living with dementia had the same for portal messages. See Tables 3 and 4 for frequencies of known contact searches.

**Figure 2:**
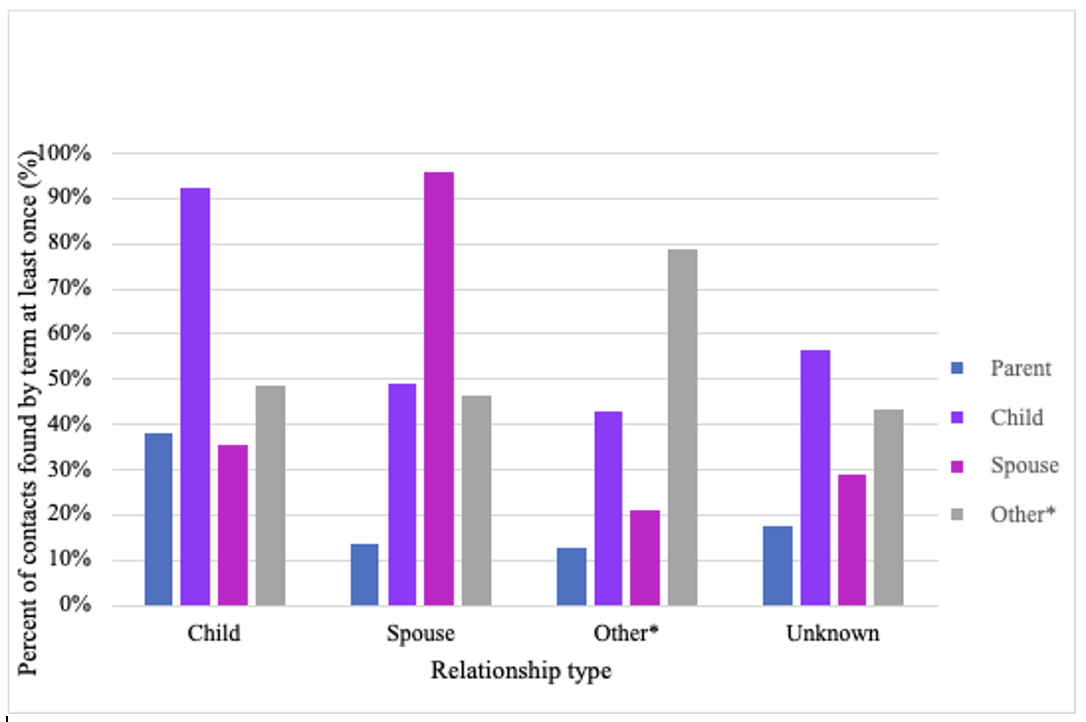
Description of caregiver terms near known contacts by caregiver type. * refers to other types of non-spousal or child relationships such as siblings, neighbors, or friends.

When comparing caregiver terms identified in the text with known relationship type (Figure 2), the caregiver term matched the correct relationship type, that is, a child-related term was noted next to the name of a known child. However, caregiver terms that did not match known relationships were also identified near known contact names. For example, while 92% of known child contacts were listed next to a child-related caregiver term, 49% of known child contacts were also listed next to other caregiver terms.

**Table 3:** Names of potential caregivers found in EHR^a^ text next to caregiver term by text type.

			Caregivers, n	Percent with one or more hit found next to caregiver terms in EHR text by text type, n (%)			
				Encounter notes	Permanent comments	Portal messages	Problem list
**Known contacts**							
	**Matched by first name**						
		Cohort^b^	789	739 (93.7)	535 (67.8)	364 (46.1)	559 (70.8)
		Contacts^c^	1667	1474 (88.4)	731 (43.9)	486 (29.2)	826 (49.6)
	**Matched by full name**						
		Cohort^b^	789	696 (88.2)	381 (48.3)	274 (34.7)	428 (54.2)
		Contacts^c^	1667	1228 (73.7)	473 (28.4)	321 (19.3)	587 (35.2)
**New names**							
	**All names found next to caregiver terms**						
		Cohort^b^	789	782 (99.2)	289 (36.6)	320 (40.6)	196 (24.8)
		Names^c^	7556	6786 (89.8)	422 (5.6)	754 (10.0)	262 (3.5)
	**Found next to caregiver term twice or more**						
		Cohort^b^	789	685 (86.8)	149 (18.9)	232 (29.4)	170 (21.5)
		Names^c^	2614	2458 (94.0)	186 (7.1)	411 (15.7)	227 (8.7)
	**Model predicted to be similar to known contacts**						
		Cohort^b^	789	692 (87.7)	263 (33.3)	176 (22.3)	195 (24.7)
		Names^c^	3706	3466 (93.5)	373 (10.1)	262 (7.1)	261 (7.0)

^a^EHR: electronic health record.

^b^Percentages represent the percent of the patient cohort with at least 1 known contact or new name found in a given text source or next to a given caregiver term at least once.

^c^Percentages represent the percent of known contacts or new names found in a given text source or next to a given caregiver term at least once.

**Table 4:** Names of potential caregivers found in EHR^a^ text by caregiver term category.

			Caregivers, n	Percent found near caregiver terms by caregiver term				
				Category, n (%)				
				Parent	Child	Spouse	Other^b^	Any
**Known contacts**								
	**Matched by first name**							
		Cohort^c^	789	363 (46.0)	628 (79.6)	447 (56.7)	559 (70.8)	745 (94.4)
		Names^d^	1667	450 (27.0)	1204 (72.2)	690 (41.4)	876 (52.5)	1490 (89.4)
	**Matched by full name**							
		Cohort^c^	789	77 (9.8)	551 (69.8)	276 (35.0)	272 (34.5)	711 (90.1)
		Names^d^	1667	82 (4.9)	916 (54.9)	375 (22.5)	381 (22.9)	1284 (77.0)
**New names**								
	**All names found next to caregiver terms**							
		Cohort^c^	789	352 (44.6)	645 (81.7)	503 (63.8)	638 (80.9)	783 (99.2)
		Names^d^	7556	719 (9.5)	3494 (46.2)	2152 (28.5)	2719 (36.0)	7556 (100)
	**Found next to caregiver term twice or more**							
		Cohort^c^	789	243 (30.8)	513 (65.0)	381 (48.3)	459 (58.2)	698 (88.5)
		Names^d^	2614	377 (14.4)	1409 (53.9)	826 (31.6)	1149 (44.0)	2614 (100)
	**Model predicted to be similar to known contacts**							
		Cohort^c^	789	173 (21.9)	641 (81.2)	3335 (42.5)	432 (54.8)	708 (89.7)
		Names^d^	3706	238 (6.4)	3389 (91.4)	624 (16.8)	908 (24.5)	3706 (100)

^a^EHR: electronic health record.

^b^Percentages represent the percent of the patient cohort with at least 1 known contact or new name found in a given text source or next to a given caregiver term at least once.

^c^Percentages represent the percent of known contacts or new names found in a given text source or next to a given caregiver term at least once.

### NLP to Identify Names of Caregivers Not Listed as Known Contacts

The NLP model identified 7556 new names in the caregiver text among 99% of the cohort. The result from our test set indicates high levels of accuracy and reliability for identifying a name (*F*_1_-score=.85; precision=.89; recall=.82). Names were most often found in the encounter notes (94%) next to a child-related caregiving term (46%). 87% of the cohort had a new name listed in their EHR caregiving text ≥2 times with a total of 2614 such names, primarily in encounter notes and patient portal messages. While new names were most often listed next to a child-related caregiving term, 44% of names listed ≥2 were listed next to “other” caregiver terms. The caregiver’s name prediction model yielded similar results, identifying 3706 potential caregiver names. The most prominent difference was that placement next to a child-related caregiving term was a strong predictor that a name was a known contact, and so 91% of the new names selected by the prediction model were found next to a child-related caregiving term. See Multimedia Appendix 3 for model results. 1620 names were selected both by occurring ≥2 times in the EHR caregiving text and by the model.

## Discussion

### Principal Results

Caregivers play a vital role in the care of people living with dementia but are often unrecognized in care [5]. As such, systematic identification of caregivers in dementia care is a national priority [4]. To address this gap, we developed a framework for merging structured and unstructured EHR data to systematically identify potential caregivers across caregiving networks. Our analysis revealed several key patterns and trends regarding the presence and distribution of caregiver-related information within structured fields and unstructured text across different sections of the EHR. These patterns highlight the prevalence of potential caregivers listed in the EHR, the location of caregiving information, and the relationship types of identified caregivers.

First, our results highlight a high prevalence of potential caregivers associated with people living with dementia. 95% of the cohort had at least 1 caregiver name listed as a known contact with an average of 2. Interestingly, a significant proportion of known contacts were also found in less conventional fields, such as the “transportation” field, underscoring the diverse sources of caregiving information within the EHR. Known contact names were also commonly found in patient portal messages. This supports previous research emphasizing that caregivers are communicating with clinicians via the portal and may serve as a venue to further support caregivers [20,21]. A large percentage (87%) of the cohort had a new name identified by the NLP model that was listed next to a caregiver term at least twice in the encounter notes and portal messages. This highlights the complexity of caregiving networks and indicates people are participating in the care of people living with dementia yet are not formally documented as known contacts. Improving standardized fields for documenting caregivers will benefit caregiver identification; however, text fields can be assessed to capture the full range of caregiving networks that are often overlooked in clinical documentation.

Our analysis of unstructured EHR text revealed ubiquitous references to caregivers across EHR locations, with caregiver terms present throughout encounter notes, patient portal messages, problem lists, and permanent comments. Current research efforts are underway to identify key EHR locations and specific encounter types where caregiver information is prominent [18,22]. In our study, permanent comments had a high yield for both names and familial keywords. Since the structured health care agent and emergency contact fields are populated into permanent comments, this represents a duplication of data, not an independent identification of possible caregivers. However, new names were also identified from the permanent comments. As such, permanent comments may be a comprehensive source of caregiving information about both known contacts and additional caregiving details.

Our study describes the relationship types of caregivers listed in the EHR. Child-related caregiving terms were most prevalent across the caregiving notes text, whereas parent-related terms (representing child or parent dyads) were more common in the patient portal messages. This aligns with the literature that children play a large part in caring for their parents, particularly those with dementia [23]. While the majority of caregivers are spouses and children (approximately 60%), many caregivers are other relatives (siblings and grandchildren; approximately 30%) or nonrelatives (10%) [24]. “Other” caregiver terms were commonly found in the permanent comments and therefore a potential resource for nontraditional caregiving information. We found a high degree of correspondence between known contact relationship types and caregiver terms, particularly within encounter notes. However, discrepancies were also observed, with caregiver terms sometimes not aligning with known relationships listed in structured fields. This emphasizes the need for improving NLP models to identify caregiver names, potential caregiving roles, and relationship types to better capture the network of caregiving, ranging from primary caregivers to extended family and friends.

The clinical benefits of rigorous methods to support caregiver identification from the EHR are compelling and vast. Such approaches provide a comprehensive view of the caregiving network. This may allow clinicians to better engage in communication and tailor caregiving interventions to various types of caregiver roles and needs. Our model could support the timely entry of people living with dementia into patient and caregiving interventions. Such timely identification is important, as caregivers report they are more likely to participate in interventions if they learn about them when they require help [25]. This work also facilitates interoperability efforts underway to link people living with dementia and caregiver records for the co-monitoring of health-related needs [11]. Linking records across the patients and caregivers would provide improved assessment and capture of both people living with dementia and caregivers’ health outcomes.

### Limitations

This research has limitations, which include a majority of White and non-Hispanic study samples from a single integrated health system, and our data only included caregivers documented in the EHR from ambulatory visits. Caregivers documented in structured fields during hospitalizations are not available. Our results are limited to documentation processes at KPCO.

In addition, we purposefully selected people living with dementia with behavioral disturbances who we considered more likely to have an informal caregiver and a caregiver documented in the EHR. However, the exclusion of patients with less severe dementia may limit the generalizability of our findings to only caregiver identification in advanced dementia. We also did not evaluate potential model performance differences by dementia subtype. Differences in clinical presentations, such as the behavioral disturbances associated with Lewy body dementia versus Alzheimer disease, represent an important avenue for future research [16]. We were also unable to confirm if the known contacts and names identified by NLP have a caregiving role in the people living with dementia’s life or consider themselves caregivers. Our methods are potentially applicable to other populations, such as individuals with less severe dementia or older adults with multiple comorbidities. However, as previous literature reports, caregiving roles are fluid and some individuals may not identify as such, especially the caregivers of people living with dementia in the early stages of illness [26,27].

Another limitation is that the NLP model used in this research may still output nonperson names in error, and any use of similar models would need to balance accuracy with breadth of results. Additionally, we did not use NLP to directly annotate and model caregivers, rather only names. This additional annotation to obtain a well-performing NLP model was not possible within the scope of this research and should be explored in future work. Although we discussed annotation processes as a team, we did not double-annotate the manual name entity. Finally, nicknames and abbreviated names are difficult to match to formal names, and we were unable to determine if newly identified names were nicknames of known contacts or patients. Any use of these methods in a clinical setting should include substantial human review. Leveraging these data sources and methods may help alert practitioners to the possibility of newly identified caregivers, but their own qualitative judgment and confirmation with patients would still be critical.

### Future Research

By integrating information from both structured and unstructured sources and leveraging NLP technologies, health care providers can gain a more comprehensive understanding of the caregiving support network surrounding people living with dementia. This holistic view can inform tailored care plans and interventions aimed at supporting both people living with dementia and their caregivers more effectively [28]. However, further research is warranted to refine NLP algorithms and validate findings across diverse health care settings to ensure the generalizability and scalability of these approaches. The extent and types of care provided by known contacts and new names remain unknown. Therefore, future analysis of text surrounding caregiver names and terms capturing caregiving roles can guide text features to improve caregiver identification in the EHR.

Our specific next steps include improving the model to identify caregiving tasks such as assistance with activities of daily living and care navigation in which identified names are provided. We will also expand the approach to include patients with less severe dementia and validate the model in multiple health systems, in particular health systems that include inpatient caregiver standardized fields.

### Conclusions

A preliminary algorithm was developed to identify potential caregivers across caregiving networks using structured and unstructured EHR data. This approach has the potential to improve health services for people living with dementia and their caregivers.

## Appendix

Multimedia Appendix 1 *ICD (International Classification of Diseases)* codes for cohort identification.

Multimedia Appendix 2 List of caregiver key terms.

Multimedia Appendix 3 Logistic regression modelling of known and new contacts.

## Abbreviations

EHR: electronic health record
HIPAA: Health Insurance Portability and Accountability Act
*ICD*: *International Classification of Diseases*
KPCO: Kaiser Permanente Colorado
NLP: natural language processing
