# Supplementary material for: Systematic Identification of Caregivers of Patients Living With Dementia in the Electronic Health Record: Known Contacts and Natural Language Processing Cohort Study
Source: J Med Internet Res. 2025 May 5;27:e63654. doi: 10.2196/63654 (PMC12089870; doi:10.2196/63654)
Supplement: Multimedia Appendix 1 [file jmir_v27i1e63654_app1.docx]

**Appendix A. ICD-Codes for Cohort Identification**

| **ICD 9** | |
| --- | --- |
| 294.11 | Dementia in conditions classified elsewhere with behavioral disturbance |
| 294.21 | Dementia, unspecified, with behavioral disturbance |
| **ICD 10** | |
| F01.51 | Vascular dementia with behavioral disturbance |
| F01.511 | Vascular dementia, unspecified severity, with agitation |
| F01.518 | Vascular dementia, unspecified severity, with other behavioral |
| F01.A1 | Vascular dementia, mild, with behavioral disturbance |
| F01.A11 | Vascular dementia, mild, with agitation |
| F01.A18 | Vascular dementia, mild, with other behavioral disturbance |
| F01.B1 | Vascular dementia, moderate, with behavioral disturbance |
| F01.B11 | Vascular dementia, moderate, with agitation |
| F01.B18 | Vascular dementia, moderate, with other behavioral disturbance |
| F01.C1 | Vascular dementia, severe, with behavioral disturbance |
| F01.C11 | Vascular dementia, severe, with agitation |
| F01.C18 | Vascular dementia, severe, with other behavioral disturbance |
| F02.81 | Dementia in other diseases classified elsewhere with behavioral disturbance |
| F02.811 | Dementia in other diseases classified elsewhere, unspecified severity, with agitation |
| F02.818 | Dementia in other diseases classified elsewhere, unspecified severity, with other behavioral disturbance |
| F02.A1 | Dementia in other diseases classified elsewhere, mild, with behavioral disturbance |
| F02.A11 | Dementia in other diseases classified elsewhere, mild, with agitation |
| F02.A18 | Dementia in other diseases classified elsewhere, mild, with other behavioral disturbance |
| F02.B1 | Dementia in other diseases classified elsewhere, moderate, with behavioral disturbance |
| F02.B11 | Dementia in other diseases classified elsewhere, moderate, with agitation |
| F02.B18 | Dementia in other diseases classified elsewhere, moderate, with other behavioral disturbance |
| F02.C1 | Dementia in other diseases classified elsewhere, severe, with behavioral disturbance |
| F02.C11 | Dementia in other diseases classified elsewhere, severe, with agitation |
| F02.C18 | Dementia in other diseases classified elsewhere, severe, with other behavioral disturbance |
| F03.91 | Unspecified dementia with behavioral disturbance |
| F03.911 | Unspecified dementia, unspecified severity, with agitation |
| F03.918 | Unspecified dementia, unspecified severity, with other behavioral |
| F03.A1 | Unspecified dementia, mild, with behavioral disturbance |
| F03.A11 | Unspecified dementia, mild, with agitation |
| F03.A18 | Unspecified dementia, mild, with other behavioral disturbance |
| F03.B1 | Unspecified dementia, moderate, with behavioral disturbance |
| F03.B11 | Unspecified dementia, moderate, with agitation |
| F03.B18 | Unspecified dementia, moderate, with other behavioral disturbance |
| F03.C1 | Unspecified dementia, severe, with behavioral disturbance |
| F03.C11 | Unspecified dementia, severe, with agitation |
| F03.C18 | Unspecified dementia, severe, with other behavioral disturbance |
