# Supplementary material for: Systematic Identification of Caregivers of Patients Living With Dementia in the Electronic Health Record: Known Contacts and Natural Language Processing Cohort Study
Source: J Med Internet Res. 2025 May 5;27:e63654. doi: 10.2196/63654 (PMC12089870; doi:10.2196/63654)
Supplement: Multimedia Appendix 2 [file jmir_v27i1e63654_app2.docx]

**Appendix B. List of Caregiver Key Terms**

| boyfriend | father | neighbor |
| --- | --- | --- |
| brother | friend | nephew |
| care companion | girlfriend | niece |
| carer | granddaughter | patient partner |
| care giver | grandson | partner |
| caregiver | guardian | POA |
| care partner | husband | Power of Attorney |
| cg | in law | protector |
| companion | in-law | proxy |
| custodian | mom | significant other |
| dad | mother | sister |
| daughter | MPOA | son |
| dtr | MDPOA | spouse |
| family | neighbor | wife |
