# Supplementary material for: Systematic Identification of Caregivers of Patients Living With Dementia in the Electronic Health Record: Known Contacts and Natural Language Processing Cohort Study
Source: J Med Internet Res. 2025 May 5;27:e63654. doi: 10.2196/63654 (PMC12089870; doi:10.2196/63654)
Supplement: Multimedia Appendix 3 [file jmir_v27i1e63654_app3.docx]

**Appendix C. Logistic Regression Modelling of Known and New Contacts**

**Table C.1. Model Variable Odds Ratios**

| **Context surrounding name** | **# Known contacts /  # Total names in context** | **Odds Ratio (95% CI)** |
| --- | --- | --- |
| Text sources |  |  |
| Found in encounter notes | 1,444 / 8,230 (17.6%) | 6.67 (4.72, 9.42) |
| Found in portal messages | 485 / 1,239 (39.1%) | 3.65 (2.91, 4.58) |
| Found in permanent comments | 728 / 1,150 (63.3%) | 11.27 (9.11, 13.95) |
| Found in problem list | 820 / 1,082 (75.8%) | 18.47 (15.17, 22.5) |
| Caregiving terms |  |  |
| Found by child term | 1,203 / 4,697 (25.6%) | 3.98 (3.29, 4.8) |
| Found by spousal term | 690 / 2,842 (24.3%) | 2.42 (2.02, 2.9) |
| Found by other term | 876 / 3,595 (24.4%) | 2.19 (1.85, 2.6) |

**Table C.2. Predicted Probability Distribution by Name Category**

| **Predicted Probability Dist. (Event="Known Contact")** | **Known Contacts (N=1,489)** | **New Names (N=7,556)** |
| --- | --- | --- |
| Mean (SD) | 0.600 (0.356) | 0.079 (0.130) |
| Minimum | 0.005 | 0.007 |
| 10th Percentile | 0.052 | 0.029 |
| 25th Percentile | 0.254 | 0.029 |
| 50th Percentile | 0.689 | 0.032 |
| 75th Percentile | 0.958 | 0.052 |
| 90th Percentile | 0.989 | 0.117 |
| Maximum | 0.995 | 0.995 |
| # Above Known Contact Percentile |  |  |
| 10th Percentile (0.052)* | 1,411 (94.8%) | **3,706 (49.1%)** |
| 25th Percentile (0.254) | 1,118 (75.1%) | 469 (6.2%) |
| 50th Percentile (0.689) | 800 (53.7%) | 117 (1.6%) |

*10^th^ percentile of known contacts used for new name selection threshold

**Table C.3. Comparison of New Names Scoring Above Model Cut-off and New Names Found in 2+ Caregiving Texts**

| **Context surrounding name** | **New names above model cut-off (n=3,706)** | **New names found in 2+ caregiving texts  (n=2,614)** |
| --- | --- | --- |
| Text sources |  |  |
| Found in encounter notes | 3,466 (93.5%) | 2,458 (94.0%) |
| Found in portal messages | 262 (7.1%) | 411 (15.7%) |
| Found in permanent comments | 373 (10.1%) | 186 (7.1%) |
| Found in problem list | 261 (7.0%) | 227 (8.7%) |
| Caregiving terms |  |  |
| Found by child term | 3,389 (91.4%) | 1,409 (53.9%) |
| Found by spousal term | 624 (16.8%) | 826 (31.6%) |
| Found by other term | 908 (24.5%) | 1,149 (44.0%) |
